# Supplementary material for: Characterization of Hormone Receptor and HER2 Status in Breast Cancer Using Mass Spectrometry Imaging
Source: Int J Mol Sci. 2023 Feb 2;24(3):2860. doi: 10.3390/ijms24032860 (PMC9918176; doi:10.3390/ijms24032860)
Supplement: Supplementary file 1 [file ijms-24-02860-s001.zip › ijms-2121609-supplementary.pdf]

Supplemental Materials

Characterization of Hormone Receptor and HER2 Status in Breast Cancer Using Mass Spectrometry Imaging

Table S1. Summary of the supervised classification ………………………………………………….. 2

Table S2. Outcome of the forward feature extraction…………………………………………………... 3

Figure S1. MS/MS fragmentation spectra of *m*/*z* 772.4 (top) and *m*/*z* 1198.7 (bottom) used for protein identification. ……………………………………………………………………………………….. 4

Figure S2. MS/MS fragmentation spectra of *m*/*z* 1428.7 (top) and *m*/*z* 1494.7 (bottom) used for protein identification. …………………………………………………………………………….……… 5

Table S3. Histopathology classification of the patient cohort. …………………………………….... 6

Table S4. Vimentin staining results for all TNBC cases of the validation sample set …………….…. 7

	Random Forest				kNN			
	ER	PR	HER2	TNBC	ER	PR	HER2	TNBC
Accuracy	0.9554	0.9223	0.9339	0.9787	0.9729	0.9522	0.9511	0.9869
95% CI	(0.9527, 0.9581)	(0.9188, 0.9258)	(0.9306, 0.937)	(0.9768, 0.9805)	(0.9707, 0.9749)	(0.9494, 0.9549)	(0.9482, 0.9538)	(0.9854, 0.9883)
P-value	$< 2.2e^{-16}$	$< 2.2e^{-16}$	$< 2.2e^{-16}$	$< 2.2e^{-16}$	$< 2.2e^{-16}$	$< 2e^{-16}$	$< 2e^{-16}$	$<2e^{-16}$
Kappa	0.8472	0.8078	0.845	0.8837	0.9098	0.885	0.8863	0.8924
Sensitivity	0.8383	0.8174	0.9576	0.9944	0.9242	0.9148	0.9624	0.9926
Specificity	0.982	0.9664	0.8818	0.8486	0.9839	0.9679	0.9261	0.9393
	**SVM**				**LDA**			
	ER	PR	HER2	TNBC	ER	PR	HER2	TNBC
Accuracy	0.9557	0.9081	0.8881	0.9879	0.9061	0.8121	0.7775	0.9591
95% CI	(0.9529, 0.9583)	(0.9043, 0.9118)	(0.884, 0.8921)	(0.9864, 0.9893)	(0.9022, 0.9098)	(0.807, 0.8171)	(0.7721, 0.7828)	(0.9565, 0.9616)
P-value	$< 2.2e^{-16}$	$< 2.2e^{-16}$	$< 2.2e^{-16}$	$< 2.2e^{-16}$	$< 2.2e^{-16}$	$< 2.2e^{-16}$	$< 2.2e^{-16}$	$< 2.2e^{-16}$
Kappa	0.8433	0.7632	0.7254	0.935	0.6509	0.4808	0.4164	0.7677
Sensitivity	0.8017	0.7271	0.9613	0.9973	0.608	0.4564	0.9314	0.9884
Specificity	0.9905	0.9841	0.7269	0.9101	0.9736	0.9614	0.4383	0.7157

Table S1. Summary of the supervised classification of the individual hormone receptor and HER status, as calculated by the respective confusion matrix.

Table S2. Outcome of the forward feature extraction form the linear discriminant analysis classification model.

ER		PR		HER2		TNBC	
m/z	Accuracy	*m/z*	Accuracy	*m/z*	Accuracy	*m/z*	Accuracy
1198.7	0.8675	1198.7	0.7652	606.1	0.7462	1198.7	0.9079
1200.7	0.8789	1200.7	0.7802	995.5	0.7483	1555.8	0.9168
911.4	0.8851	1555.8	0.7857	842.5	0.7511	1584.8	0.9222
805.4	0.8934	1104.6	0.7904	882.5	0.7542	674.3	0.9301
1104.6	0.896	1584.8	0.7959	688.4	0.7567	1094.6	0.9353

Figure S1. MS/MS fragmentation spectra of *m/z* 772.4 (top) and *m/z* 1998.7 (bottom) used for protein identification.

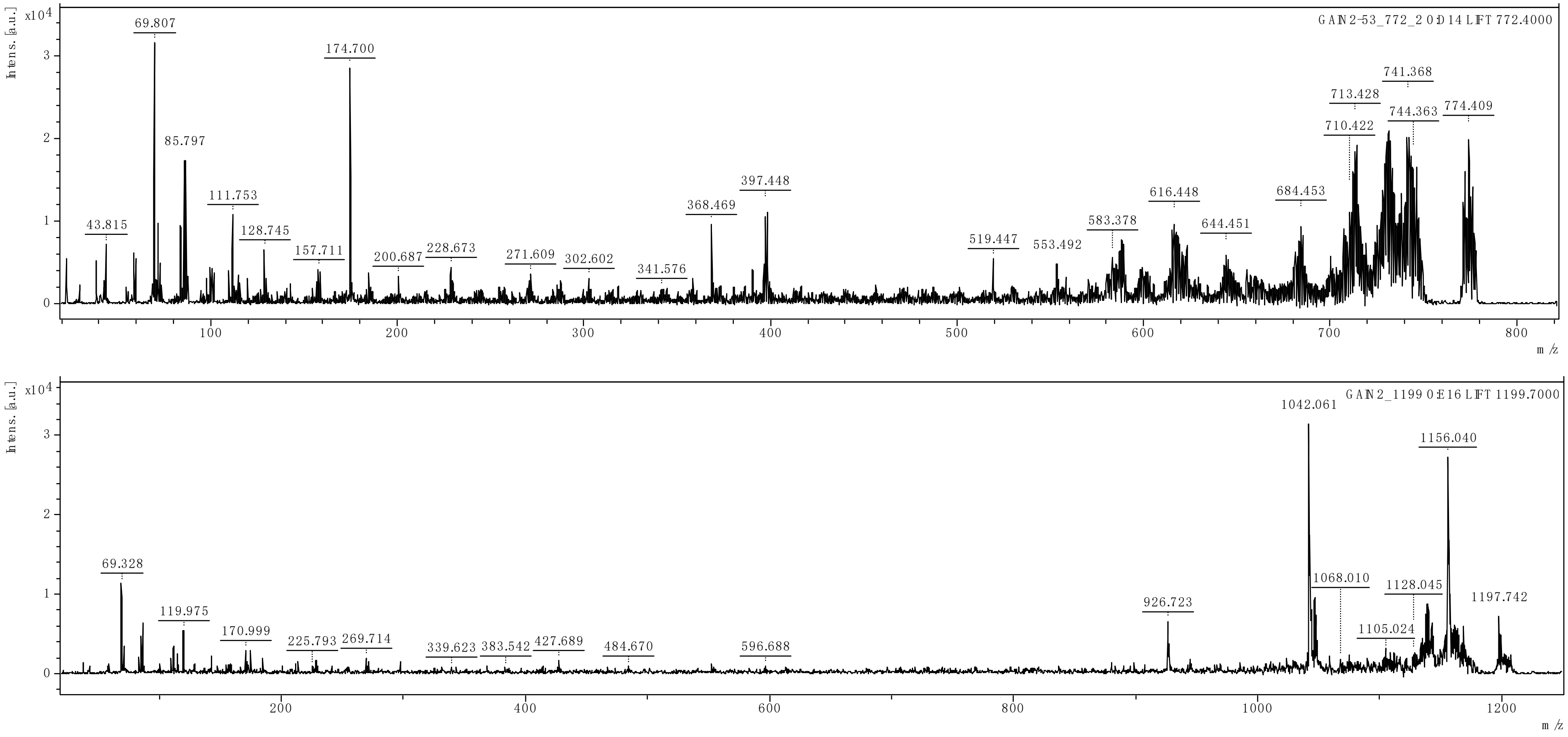

Figure S2. MS/MS fragmentation spectra of *m/z* 1428.7 (top) and *m/z* 1494.7 (bottom) used for protein identification.

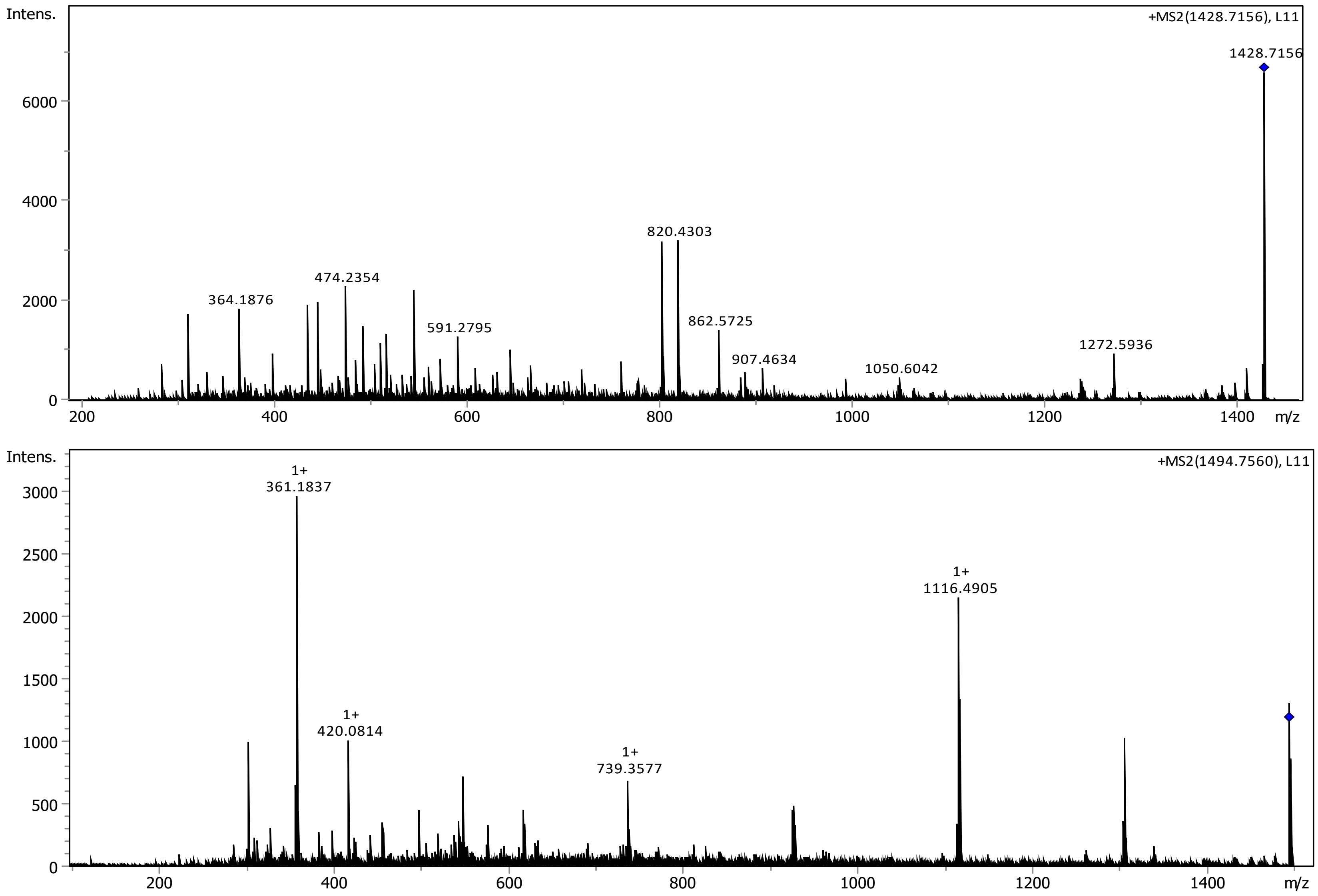

Parameter	GAIN-1 cohort	Study cohort	p-value	MSI cohort
	n(%)	n(%)		n (%)
**Age (years)**				
Mean, range	50 (20-72)	50 (23-71)	0.1122	
< 40	438 (14.6)	194 (14.7)	0.2443	
40-49	1057 (35.5)	488 (14.7)		
50-59	968 (32.3)	418 (31.7)		
≥60	531 (17.7)	218 (16.5)		
**Surgery**			0.2354	
BCS	1672 (55.9)	753 (57.1)		
Mastectomy	1320 (44.1)	565 (42.9)		
Unkonown	2	0		
**Chemotherapy**			0.7967	
EPC	1500 (50.1)	664 (50.4)		
EC-PwX	1494 (49.9)	654 (49.6)		
**Local ER**			0.8345	**Re-evaluated ER**
Positive	2206 (73.7)	974 (73.9)		850 (80.7)
Negative	787 (73.7)	344 (26.1)		203 (19.3)
Unknown	1	0		32
**Local PR**			0.5831	**Re-evaluated PR**
Positive	2011 (67.2)	893 (67.8)		739 (69.9)
Negative	982 (32.8)	425 (32.2)		318 (30.1)
Unknown	1	0		28
**Local HER2**			0.7957	**Re-evaluated HER2**
Positive	724 (24.8)	321 (25.1)		369 (34.6)
Negative	2191 (75.2)	959 (74.9)		696 (65.4)
Unknown	79	38		20
**Local subtypes**			0.5372	**Subtypes**
Luminal A	1223 (41.8)	542 (42.0)		596 (58.3)
Luminal B	1031 (35.2)	465 (36.0)		121 (11.8)
ER-/PR-/HER2+	254 (8.7)	102 (7.9)		200 (19.6)
TNBC	421 (14.4)	182 (14.1)		105 (10.3)
Unknown	65	27		63
**Histologic type**			0.2022	
Ductal (NST)	2314 (77.3)	1023 (77.6)		
Lobular	374 (12.5)	151 (11.5)		
Other	306 (10.2)	144 (10.9)		
**Tumor grade**				
G1	96 (3.2)	44 (77.6)		
G2	1507 (50.4)	649 (49.3)		
G3	1385 (46.4)	624 (47.4)		
Unknown	6	1		
**Tumor stage**			0.9392	
pT1	955 (32.0)	423 (32.2)		
pT2	1669 (55.9)	738 (49.3)		
pT3	305 (10.2)	130 (9.9)		
pT4	55 (1.8)	23 (1.8)		
Unknown	10	4		
**Nodal stage**			0.0273	
pN1	1131 (37.8)	533 (40.4)		
pN2	1058 (35.3)	449 (34.1)		
pN3	805 (26.9)	336 (25.5)		

Table S3. Histopathology classification of the patient cohort. GAIN-1 corresponds to the larger cohort from which the study cohort was obtained, with the respective local characterization. The study cohort was measured with mass spectrometry imaging (MSI). However, to avoid inter-observer bias, two pathologists re-evaluated the study cohort (with the exception of lost cores). This classification (summarized in the last column) was used as the basis for training of classification models.
EC-PwX, epirubicin, cyclophosphamide, paclitaxel and capecitabine; EPC, epirubicin, paclitaxel and cyclophosphamide; TNBC, triple negative breast cancer; ER, Estrogen receptor; GAIN, German Adjuvant Intergroup Node; PR, progesterone receptor; HER2, human epidermal growth factor receptor 2; BCS, breast conserving therapy; NST, no special type.

Table S4. Vimentin staining results for all TNBC (n=51) cases of the validation sample set, specified for tumor subtype. NST – no special type.

Vimentin staining	n (%)	Tumor subtype	n
Strong positive	17 (33.3)	NST, G3	14
		Medullary, G3	2
		Metaplastic, G3	1
Medium positive	15 (29.4)	NST, G3	9
		NST, G2	3
		Medullary, G3	3
Weak positive	8 (15.7)	NST, G3	4
		Medullary, G3	2
		Lobular, G3	1
		Ductulolobular, G3	1
Negative	11 (21.6)	NST, G3	4
		Lobular, G2	1
		NST, G2	1
		NST, G1	1
		Medullary, G3	1
		Apocrine, G3	1
		Papillary, G2	1
		Ductulolobular, G3	1
